# The mechanism of auxin driving *Xanthium strumarium* invasion

**DOI:** 10.3389/fpls.2025.1705498

**Published:** 2025-11-27

**Authors:** Chang Zhang, Yuan Cheng, Hai-Long Wu, Xiao Han, Yu-Xuan Du, Ting-Ting Zhao, Fa-Zhao Qi, Yu-Long Feng

**Affiliations:** Liaoning Key Laboratory for Biological Invasions and Global Changes, College of Bioscience and Biotechnology, Shenyang Agricultural University, Shenyang, Liaoning, China

**Keywords:** biological invasion, hormone-driven hypothesis, auxin, growth defense strategy, omics analysis

## Abstract

Biological invasion is a critical ecological challenge, exerting profound impacts on ecosystem stability, public health, and economic sustainability. To better understand the successful invasion mechanism, many hypotheses have been proposed. However, the roles of hormones in it are not clear, especially for the differential effects of hormones on invasive and native plants, and its mechanisms. In this study, we hypothesized that there was not only a difference in hormone (auxin) concentration between invasive and native plants but also a difference in the effect of auxin on invasive and native plants. The above characteristics drove the successful invasion of invasive plants. To verify this hypothesis and investigate the mechanism, auxin concentration, and physiological index determination, transcriptomic and metabolomic analyses were performed. Our results showed that the auxin concentration was higher in invasive plants *Xanthium strumarium* and the growth-promoting effect of auxin on the invader was stronger than its native congener *X. sibiricum*. Compared with *X. sibiricum*, the auxin signal transduction in *X. strumarium* was more strongly activated, and more genes were differentially expressed in response to auxin. Auxin strongly promoted the growth of *X. strumarium* by enhancing photosynthesis, reducing the resources investment in defense and stress resistance, and promoting cell growth and division. However, the promoting effect of auxin on *X. sibiricum* was mainly achieved by enhancing photosynthesis. Our results elucidated the mechanism of auxin driving *X. strumarium* invasion, which contributed to the systematic proposal of the hormone-driven hypothesis.

## Introduction

Biological invasion is a global ecological problem, which seriously threatens the ecosystems, human health, and economic development (Hulme, 2021; Roy et al., 2024; Wang et al., 2021). Biological invasion has been estimated to cause an economic loss of trillions of dollars (Diagne et al., 2021). The numbers of alien species will continue to grow rapidly, and biological invasion will become increasingly serious (Seebens et al., 2021). To better understand the successful invasion mechanism, many hypotheses have been proposed (Gioria et al., 2023). However, the roles of hormones in biological invasion are not clear. The relevant hypotheses need to be systematically proposed.

Compared with native plants, invasive plants often have stronger growth advantages, which have a positive impact on their successful invasion (Gioria et al., 2023; Kawawa Abonyo and Oduor, 2024; Sun et al., 2025). Recent studies have shown that the competitive growth advantage of invasive species over native species is usually associated with differential concentration of some hormone (Manoharan et al., 2019; Shan et al., 2024; Zhang et al., 2024). The concentrations of abscisic acid (ABA) and the resistance ability to drought stress were higher in invasive plant *Sphagneticola trilobata* than its native plant *S. calendulacea*, promoting its invasion in drought habitats (Zhang et al., 2024). The concentrations of jasmonic acid (JA), salicylic acid (SA), and ethylene and the resistance ability to pathogen *Rhizoctonia solani* were higher in invasive plant *Alternanthera philoxeroides* than its native plant *A. sessilis* during pathogenesis, promoting its invasion in the environments infected by pathogens (Manoharan et al., 2019). In addition, a study based on five pairs of invasive and native plants showed that invasive plants had higher gibberellin3 (GA3) concentration and higher total biomass compared with their native plants, promoting their invasion (Shan et al., 2024). However, few studies have focused on the differences in auxin concentration between invasive and native species. Moreover, the mechanisms of auxin driving plant invasion are still largely unknown.

Auxin is an important growth-promoting hormone and plays important roles in multiple physiological processes related to growth and development (Jeong et al., 2009; Jin et al., 2023; Li et al., 2023; Yuan et al., 2019; Zhou et al., 2024). Exogenous application of auxin significantly promoted the growth of *Syringa villosa*, increasing height, stem diameter, leaf area, and photosynthetic rate (Jin et al., 2023). Activation of the auxin signaling pathway significantly increased chlorophyll concentration, photosynthetic rate, starch accumulation and soluble sugars in tomatoes (Yuan et al., 2019). Auxins may also have impacts on these physiological processes of invasive plants, thereby promoting growth and invasion. It is reported that the concentrations of auxin were significantly higher in the turning part of the stem than those in the upper part of the stem when invasive plants *Mikania micrantha* climbed trees, which contributes to quickly covering trees and promoting invasion (Chen et al., 2024). Compared with growing alone, the concentrations of auxin were significantly higher in the leaves of *Flaveria bidentis* when it competed with native plants, activating the auxin signaling pathway and promoting its rapid growth (Zhang et al., 2025). However, the specific physiological processes through which auxin primarily promotes invasion and the corresponding mechanisms remain unknown.

Previous studies have shown that auxin has different effects on different varieties of corn, making some corn varieties more competitive (Zhou et al., 2024). In addition, the concentrations of some other hormones, such as GA, JA, and SA, were also differences between invasive and native species, promoting the invasion of invasive species (Manoharan et al., 2019; Shan et al., 2024; Zhang et al., 2024). Although there are few studies on auxin concentration and effect in invasive plants, similar results may be found. In this study, we hypothesize that compared with native plants, the concentration of auxin is higher in invasive plants and the growth-promoting effect of auxin on invasive plants is stronger, driving its successful invasion. To test this hypothesis and further elucidate its mechanism, invasive plant *Xanthium strumarium* and its native congener *X. sibiricum* were used. We first measured the concentration of auxin in the two *Xanthium* species and then determined the response of growth- and photosynthesis-related indicators in the two *Xanthium* species to auxin. Subsequently, transcriptomic and metabolomic analyses were conducted to further elucidate the mechanism by which auxin drove *X. strumarium* invasion. Finally, we integrated the results obtained from this study and other studies on hormone-driven invasion of alien plants and systematically proposed the hormone-driven hypothesis, which provided a new perspective for biological invasion.

## Materials and methods

### Materials and growth conditions

*X. strumarium* is a noxious invasive plant, which has caused serious harm to crop yield and animal husbandry, and widely distributed in Northern China. In this study, the seeds of *X. strumarium* and its native congener *X. sibiricum* were collected, stored, and disinfected as those described in Luo et al. (2022). Disinfected seeds were germinated in Petri dishes with wet filter papers in the greenhouse with 28 °C and the photoperiod of 16/8 h (16,000 Lux day/dark night). After approximately 3 days, the seeds were germinated and transferred to flower pots filled with vermiculite. *X. strumarium* and *X. sibiricum* were mixed for cultivation (each flower pot contained one *X. strumarium* and one *X. sibiricum*). Distilled water was poured every 2 days to keep the vermiculite moist. On the 7th day after germination, distilled water was replaced with 1/2 Hoagland nutrient solution and maintained at a frequency of once every 2 days. One week later, parts of *X. strumarium* and *X. sibiricum* were sprayed with 10 mg L^−1^ IAA on their leaves. Other *X. strumarium* and *X. sibiricum* were sprayed with distilled water on their leaves. IAA or water was sprayed every 3 days for 1 week, when the promoting effect of auxin on the growth of *X. strumarium* has become apparent. After that, *X. strumarium* and *X. sibiricum* treated with and without IAA were sampled for the determination of the following indicators and omics analysis.

### Growth and photosynthetic indicator determination

The aboveground part is the main site for photosynthesis, and the establishment of aboveground parts’ growth advantages in invasive plants has positive impacts on their successful invasion, which requires more attention. To compare the differences in the promotion effects of auxin on aboveground growth of *X. strumarium* and *X. sibiricum*, height, stem diameter, leaf area, aboveground biomass, and photosynthetic rate were measured, which were described in Zhang et al. (2023). The auxin response indexes based on the above indicators were calculated using the following formula: ((index values of each biological replicate under auxin treatment/the average index values of each biological replicate in the control group) −1) × 100%. Six independent biological replicates were used.

### IAA concentration determination

The aboveground parts of *X. strumarium* and *X. sibiricum* treated without IAA were used for IAA concentration determination. Three independent biological replicates were used for the IAA concentration determination in each of the two *Xanthium* species. Samples were ground with PBS (pH=7.2-7.4, 0.01 M), and the supernatant was added to the enzyme-linked immunosorbent assay (ELISA) package, followed by incubation, washing, addition of enzyme-labeled reagent, reincubation, rewashing, color reaction, termination, and measurement of OD_450_, according to the instructions of the plant IAA ELISA kit (mmbio, Yanchen, China). The concentrations of IAA can be calculated based on the OD_450_ value using a standard curve with R² value 0.999 (**Supplementary Figure S1**).

### Transcriptomic analysis

The aboveground parts of *X. strumarium* and *X. sibiricum* treated with and without IAA were used for transcriptomic analysis. Three independent biological replicates were used for each treatment of *X. strumarium* and *X. sibiricum.* Samples were ground with liquid nitrogen, and total RNA was extracted by the MiniBEST Universal RNA Extraction Kit (Takara, Dalian, China). The integrity, quality, and concentration of total RNA were checked by Agilent 2100 Bioanalyzer (Agilent Technologies, Palo Alto, USA), 1% agarose gel electrophoresis, and NanoDrop (Thermo Fisher Scientific, Inc., Waltham, USA), respectively. After passing the test, RNA sequencing was carried out according to the method described in Zhang et al. (2023). Briefly, 3 μg total RNA was used to generate sequencing libraries by the TruSeq RNA Sample Preparation Kit (Illumina, San Diego, USA). The obtained mRNA was purified and fragmented by poly-T oligo-attached magnetic beads and divalent cations, respectively, which were used for the synthesis of first-strand cDNA. After that, second-strand cDNA was also synthesized, followed by end repair, dA tailing, and adaptor ligation. The AMPure XP system (Beckman Coulter, Beverly, CA, USA) was used to select 400-500-bp cDNA fragments which were used for PCR reaction with Illumina PCR Primer Cocktail in 15 cycles. Qualified PCR products were loaded on a NovaSeq 6000 platform (Illumina) by Shanghai Personal Biotechnology Cp. Ltd.

After filtering the raw data obtained from the NovaSeq 6000 platform (Illumina), clean data were used to assemble transcripts through Trinity (v2.5.1) software. Functional annotation of these obtained transcripts was performed using multiple databases, including NR, GO, KEGG, Swiss-Prot, and Pfam. Gene expression levels were calculated by RSEM (1.2.31). Differentially expressed genes (DEGs) were selected by |log_2_ fold change| > 1 and *P* value < 0.05. The GO terms and KEGG pathway enrichment analyses of DEGs were performed by topGO and clusterProfiler packages in R, respectively.

### Metabolomic analysis

The aboveground parts of *X. strumarium* and *X. sibiricum* treated with and without IAA were used for metabolomic analysis. Five independent biological replicates were used for each treatment of *X. strumarium* and *X. sibiricum.* Samples were homogenized by shaking in the mixed extraction solution with methanol: acetonitrile: water = 2: 2:1 (V/V), and ice bath ultrasound was used to extract metabolites. QC was added to the extraction solution to evaluate instrument stability and data reliability. Metabolome determination and analysis referred to the methods described in Alseekh et al. (2021).

Metabolites in the supernatant were separated on the Vanquish UHPLC system (Thermo Fisher Scientific, Waltham, MA, USA) equipped with ACQUITY UPLC HSS T3 (100Å, 1.8 µm, 2.1 mm × 100 mm). The relevant parameter settings were as follows: injection volume, 2 μL; flow rate, 0.4 mL min^−1^; column temperature, 40 °C. The mobile phases were 0.1% formic acid aqueous solution (phase A) and acetonitrile solution containing 0.1% formic acid (phase B). The gradient program of phase A/phase B was 95: 5 (v/v) at 0 min, 95: 5 (v/v) at 1 min, 5: 95 (v/v) at 4.7 min, 5: 95 (v/v) at 6 min, 95: 5 (v/v), at 6.1 min and 95: 5 (v/v) at 8.5 min.

After metabolites were separated by UPLC, mass spectrometry data of metabolites in positive and negative ion modes were collected using the HESI source in the Thermo Orbitrap Exploris 120 mass spectrometer (Thermo Fisher Scientific, Waltham, MA, USA). The relevant parameter settings were as follows: spray voltage, 3.5 kV/−3.0 kV; sheath gas, 40 arb; auxiliary gas, 10 arb; capillary temperature, 320°C; auxiliary gas temperature, 300°C, primary resolution, 60,000; scanning range, 70-1,000 m/z; dynamic removal time, 4 s; secondary resolution, 15,000; HCD collision energy, 30%.

Finally, metabolites were identified based on multiple databases, including mzCloud, Lipid Maps, HMDB, and massbank. The XCMS package in R was used to calculate the abundance values of metabolites, which can reflect metabolite concentrations. Differential metabolites were selected by |log_2_ fold change| > 1, *P* value < 0.05 and vip value > 1. The Pheatmap package in R was used to visualize the abundance values of differential flavonoids under auxin treatment in *X. strumarium* and *X. sibiricum*.

### Statistical analysis

The significance of the difference was calculated by independent sample t-test using SPSS version 24 (SPSS, Inc., Chicago, IL, USA).

## Results

### The differences in growth-promoting effect of auxin and auxin concentrations between *X. strumarium* and *X. sibiricum*

**Figure 1:**
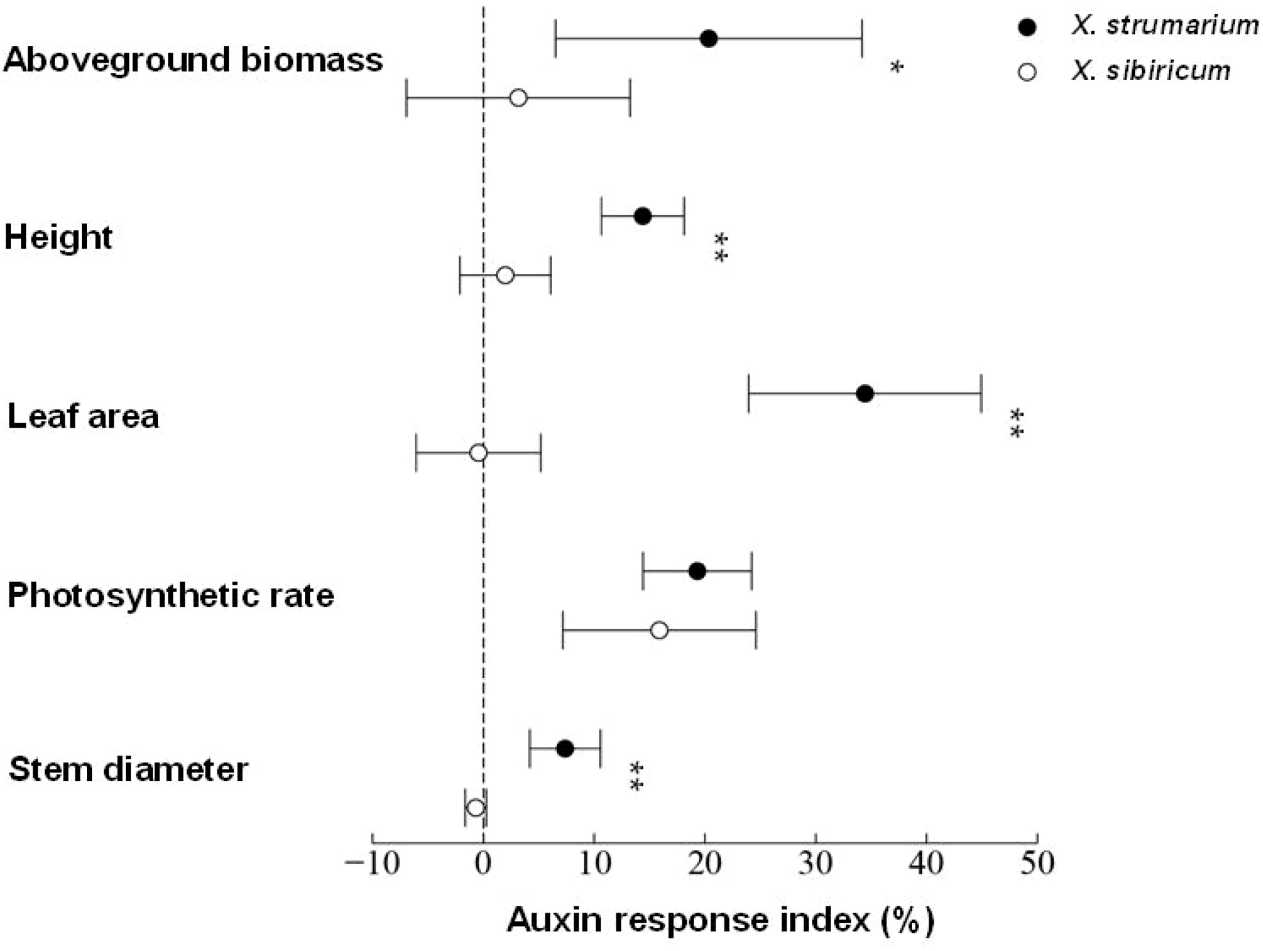
Auxin response indexes of aboveground biomass, height, leaf area, photosynthetic rate, and stem diameter in *X. strumarium* and *X. sibiricum*. The auxin response index can be used to compare the differences in the promotion effects of auxin on growth of *X. strumarium* and *X. sibiricum*. Taking aboveground biomass of *X. strumarium* as an example, the auxin response index was calculated using the following formula: ((aboveground biomass of each biological replicate of *X. strumarium* under auxin treatment/the average aboveground biomass of *X. strumarium* in the control group) −1) × 100%. Mean ± SE (n = 6). * and ** represent the auxin response indexes were significantly higher in *X. strumarium* than those in *X. sibiricum* with *P* < 0.05 and <0.01, respectively.

**Figure 2:**
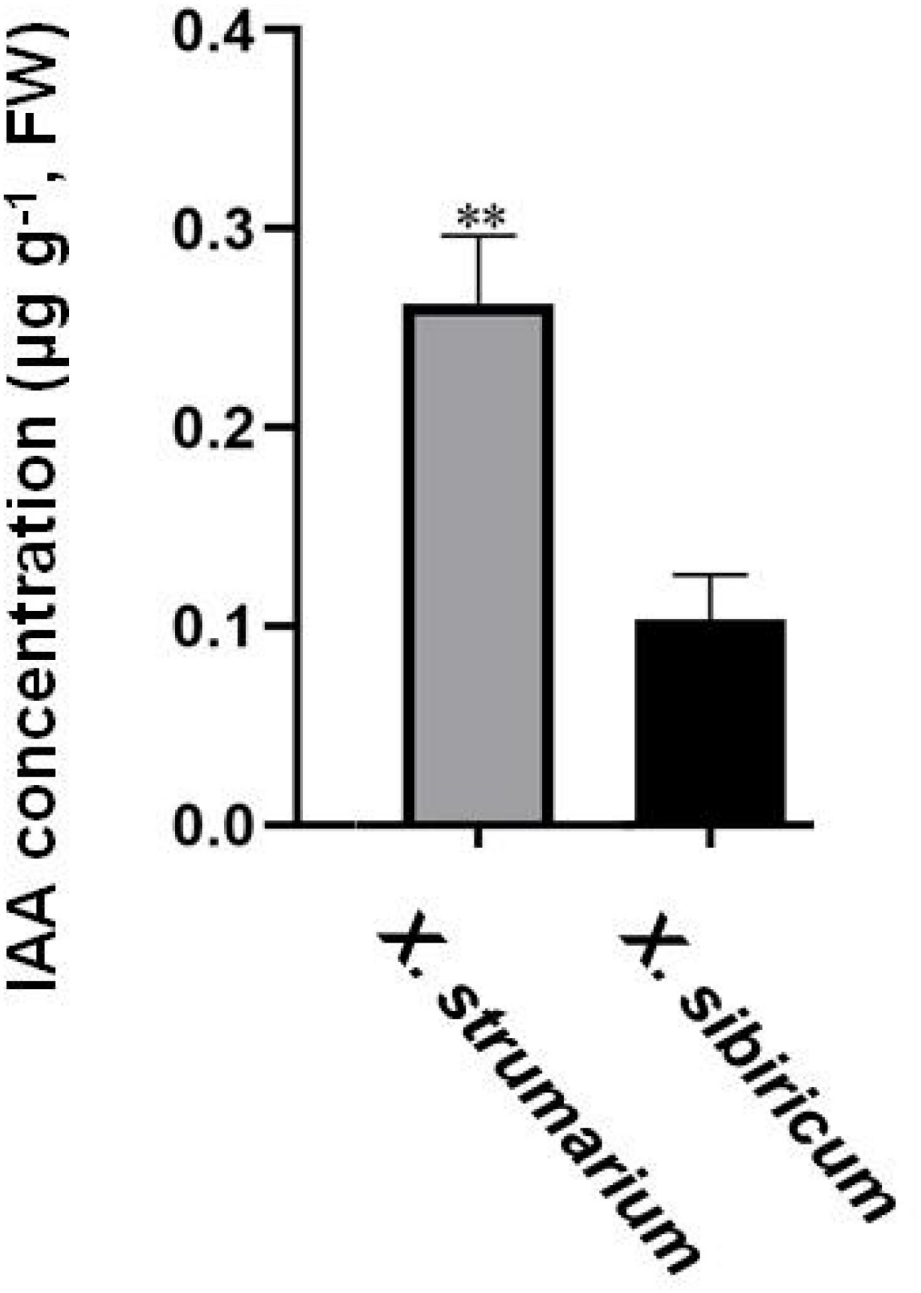
IAA concentrations in the aboveground of *X. strumarium* and *X. sibiricum*. Mean ± SE (n = 3). ** represents IAA concentration was significantly higher in *X. strumarium* than that in *X. sibiricum* (*P* < 0.01).

Auxin positively promoted the growth of invasive plant *X. strumarium* (**Figure 1**). Exogenous application of auxin increased the aboveground biomass, height, leaf area, photosynthetic rate, and stem diameter by 20.35%, 14.39%, 34.45%, 19.31%, and 7.38% in *X. strumarium*, respectively. However, the growth-promoting effect of auxin on its native congener *X. sibiricum* was limited. Exogenous application of auxin only increased the aboveground biomass, height, and photosynthetic rate by 3.16%, 1.98%, and 15.89% in *X. sibiricum*, respectively. Exogenous application of auxin hardly increased the leaf area and stem diameter in *X. sibiricum*. Except for photosynthetic rate, the promoting effects of the same-level auxin on other indicators detected in this study were significantly higher in *X. strumarium* than those in *X. sibiricum*. Moreover, auxin concentrations were significantly higher in *X. strumarium* than that in *X. sibiricum* (**Figure 2**). Compared with *X. sibiricum*, the stronger growth-promoting effect of auxin on *X. strumarium* and the higher auxin concentration in *X. strumarium* contribute to the successful invasion of *X. strumarium.*

### The genes expression responses to auxin in *X. strumarium* and *X. sibiricum*

**Figure 3:**
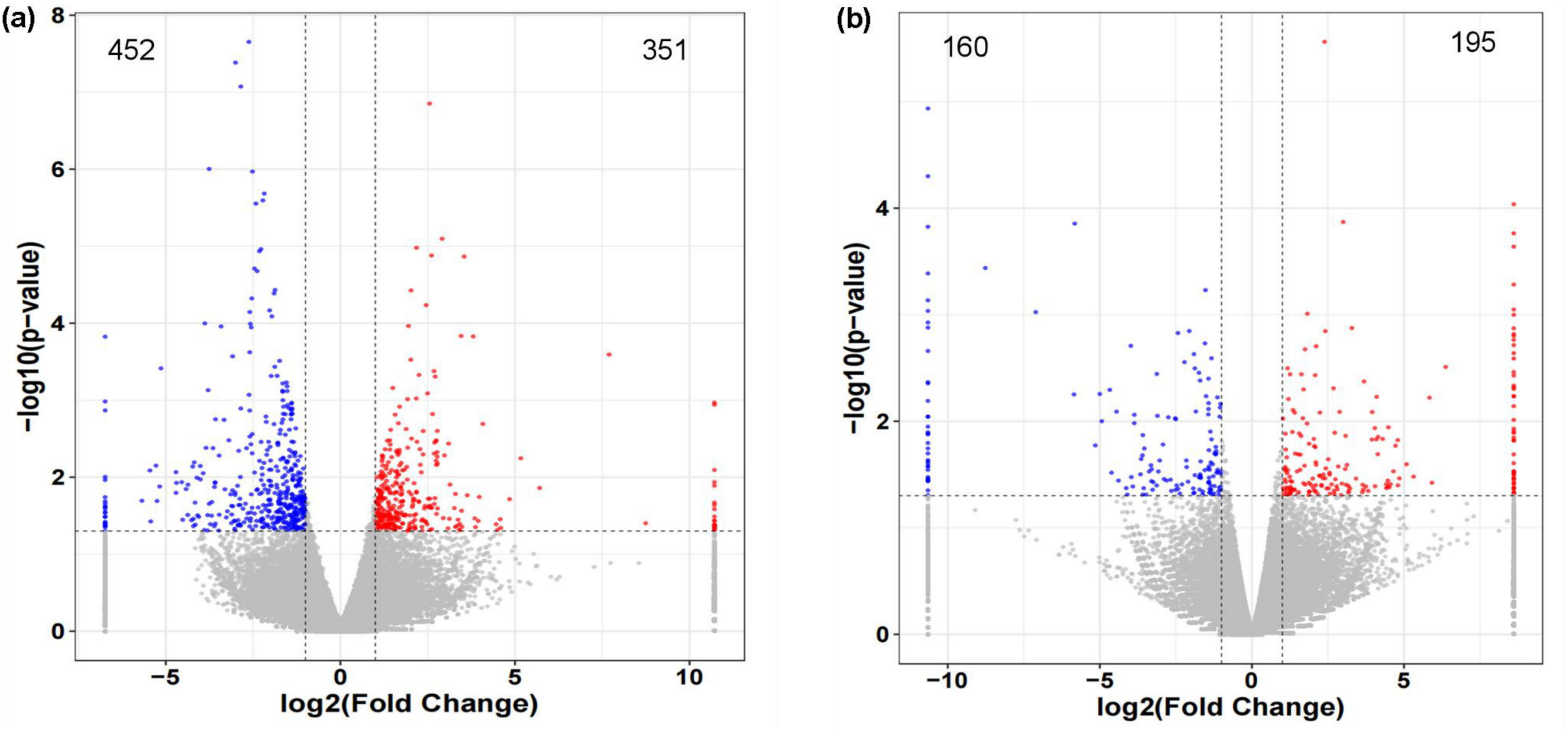
Volcano maps of differentially expressed genes (DEGs) between auxin treatment and control group in the aboveground of *X. strumarium***(a)** and *X. sibiricum***(b)**. The red, blue, and gray dots indicate significant induction, significant inhibition, and no significant difference under auxin treatment, respectively.

To investigate the mechanism of auxin promoting the growth of *X. strumarium*, and compare the mechanisms of differential response to auxin between *X. strumarium* and *X. sibiricum*, transcriptome sequencing analysis was performed. The numbers of sequencing reads and bases for all samples ranged from 4.07 ×10^7^ to 7.16 ×10^7^ and 6.14 ×10^9^ to 1.08 ×10^10^, respectively (**Supplementary Table S1**). Q20 and Q30 values for all samples were all above 98% and 96%, respectively. N% for all samples were less than 0.11%. Transcriptome sequencing data quality meets analysis requirements. There were 803 genes significantly differentially expressed in *X. strumarium* between auxin treatment and control (**Figure 3**). Among them, 351 and 452 genes were significantly induced and inhibited by auxin, respectively. However, there were only 355 differentially expressed genes (DEGs) in *X. sibiricum* between auxin treatment and control. A total of 195 genes of them were induced by auxin, and others were inhibited by auxin. *X. strumarium* had a stronger response to auxin compared to *X. sibiricum*.

**Figure 4:**
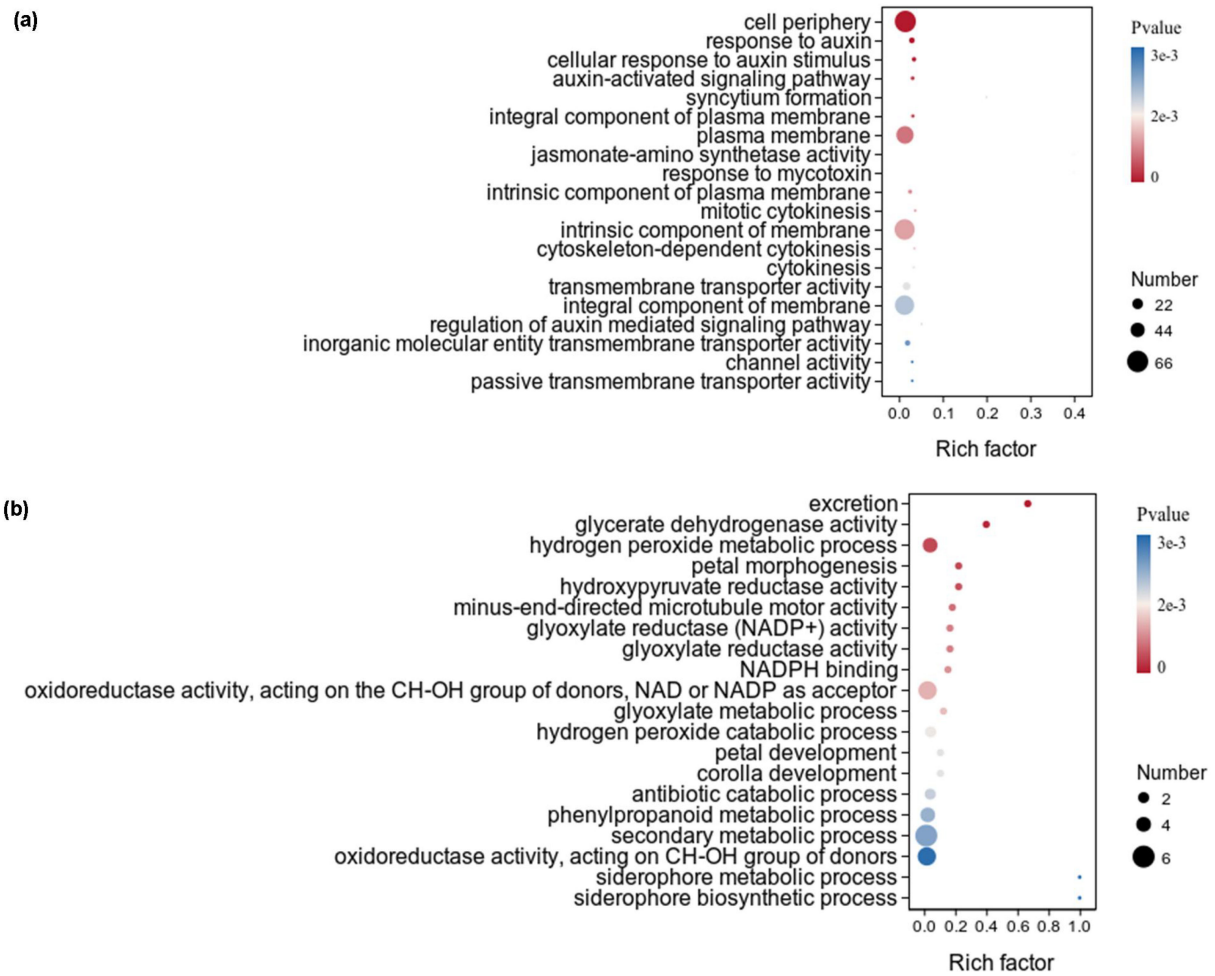
GO term enrichment analysis of upregulated DEGs under auxin treatment in the aboveground of *X. strumarium***(a)** and *X. sibiricum***(b)**. The top 20 GO terms ranked by significance were listed. Different colors represent different *P* values, increasing from red to blue. The sizes of the circle represent the numbers of upregulated DEGs enriched into different GO terms. Rich factors represent the proportions of upregulated DEGs enriched to all genes in this GO terms.

The upregulated DEGs of *X. strumarium* were significantly enriched in 187 GO terms, and the top 20 GO terms ranked by significance were listed (**Figure 4a**). Among the top 20 GO terms, the significance and numbers of DEGs enriched in cell periphery were the highest. The rich factors of DEGs enriched in top 20 GO terms are basically the same. There were six, four, four, and three GO terms respectively related to cellular components, auxin, transport activity, and cytokinesis. However, none of the top 20 GO terms which upregulated DEGs of *X. sibiricum* significantly enriched in was related to cellular components, auxin, transport activity, and cytokinesis (**Figure 4b**). Among the top 20 GO terms which upregulated DEGs of *X. sibiricum* significantly enriched in, there were eight GO terms related to biosynthetic, metabolic, and catabolic processes, although the numbers of DEGs enriched in these GO terms were very small. Six and three GO terms respectively related to enzymatic activity and flower. The significance and rich factors of DEGs enriched in excretion were the highest. The numbers of DEGs enriched in secondary metabolic process were the highest.

**Figure 5:**
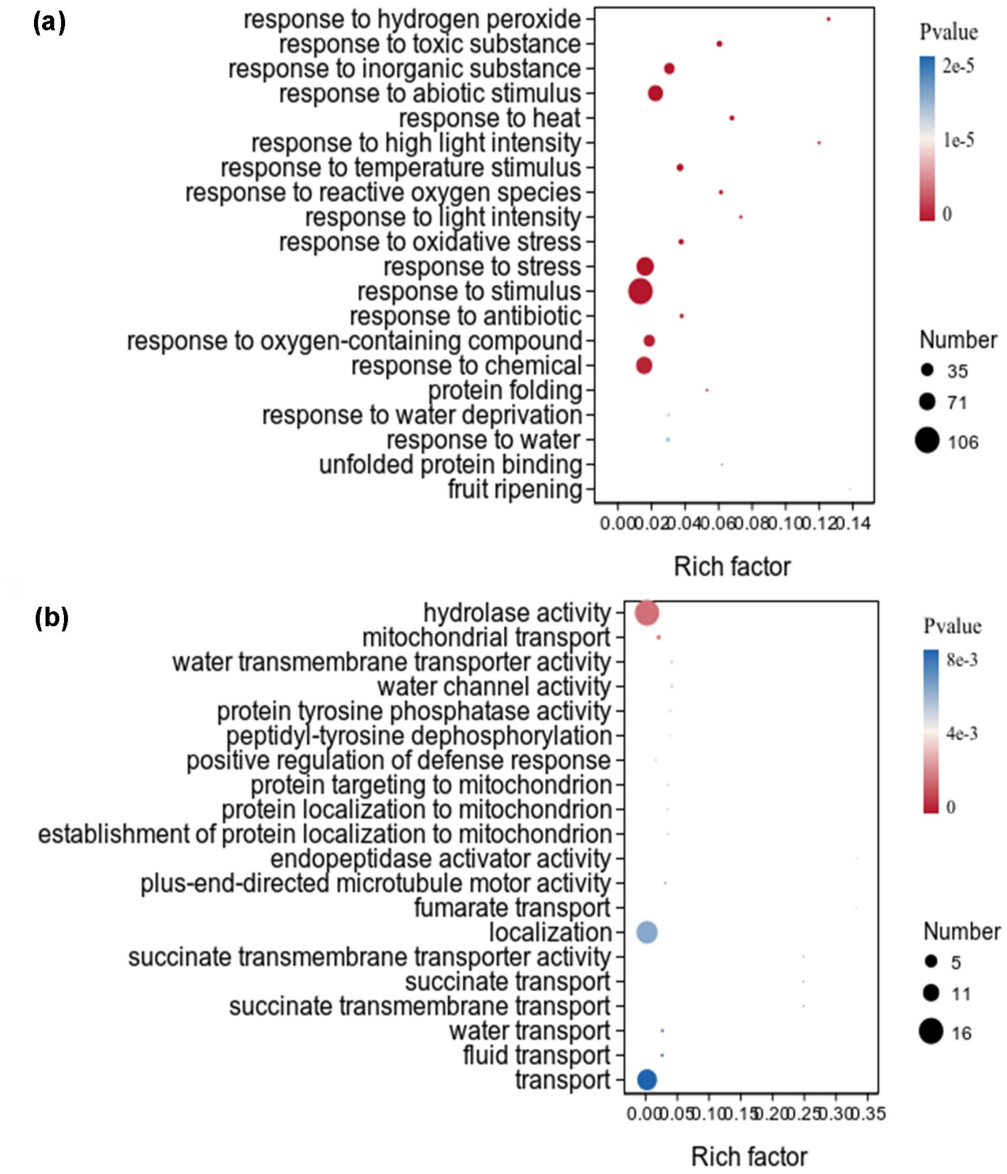
GO term enrichment analysis of downregulated DEGs under auxin treatment in the aboveground of *X. strumarium***(a)** and *X. sibiricum***(b)**. The top 20 GO terms ranked by significance were listed. Different colors represent different *P* values, increasing from red to blue. The sizes of the circle represent the numbers of downregulated DEGs enriched into different GO terms. Rich factors represent the proportions of downregulated DEGs enriched to all genes in this GO terms.

The downregulated DEGs of *X. strumarium* were significantly enriched in 446 GO terms, and the top 20 GO terms ranked by significance were listed (**Figure 5a**). Among the top 20 GO terms, the significance and rich factor of DEGs enriched in response to hydrogen peroxide were the highest. The number of DEGs enriched in response to stimulus was the highest. Most of these top 20 GO terms were related to stress response. However, only one GO term in the top 20 GO terms which downregulated DEGs of *X. sibiricum* significantly enriched in was related to stress response (**Figure 5b**). Among the top 20 GO terms which downregulated DEGs of *X. sibiricum* significantly enriched in, hydrolase activity had the highest DEGs enrichment significance and numbers. The rich factors of DEGs enriched in top 20 GO terms are basically the same.

**Figure 6:**
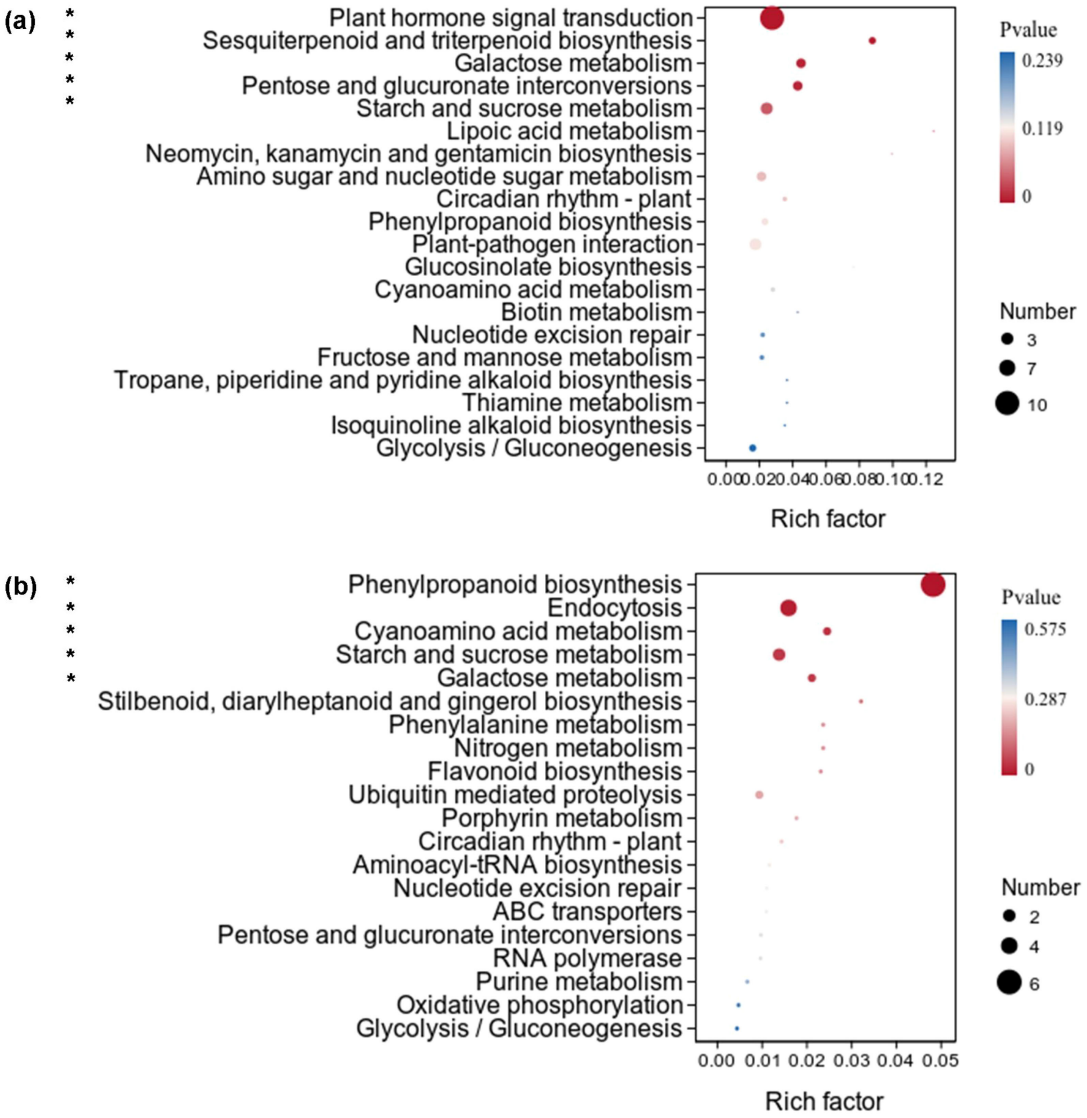
KEGG pathway enrichment analysis of upregulated DEGs under auxin treatment in the aboveground of *X. strumarium***(a)** and *X. sibiricum****(*b*)***. The top 20 KEGG pathways ranked by significance were listed. * indicates that upregulated DEGs were significantly enriched in this KEGG pathway (*P* < 0.05). Different colors represent different *P* values, increasing from red to blue. The sizes of the circle represent the numbers of upregulated DEGs enriched into different KEGG pathways. Rich factors represent the proportions of upregulated DEGs enriched to all genes in this KEGG pathway.

The upregulated DEGs of *X. strumarium* were significantly enriched in five KEGG pathways, namely, plant hormone signal transduction, sesquiterpenoid and triterpenoid biosynthesis, galactose metabolism, pentose and glucuronate interconversions, and starch and sucrose metabolism (**Figure 6a**). The significance and numbers of DEGs enriched in plant hormone signal transduction were the highest. Many auxin signal transduction-related genes were significantly induced by auxin (**Supplementary Figure S2**), which enhanced the response of *X. strumarium* to auxin. The rich factors of DEGs enriched in sesquiterpenoid and triterpenoid biosynthesis was the highest (**Figure 6a**). Among the five KEGG pathways which upregulated DEGs of *X. strumarium* were significantly enriched in, there were three KEGG pathways belonging to carbohydrate metabolism, which contributed to enhancing photosynthesis. There were also five KEGG pathways which upregulated DEGs of *X. sibiricum* were significantly enriched in, and three of them belonged to carbohydrate metabolism (**Figure 6b**). Phenylpropanoid biosynthesis had the highest DEG enrichment significance, numbers, and rich factors.

**Figure 7:**
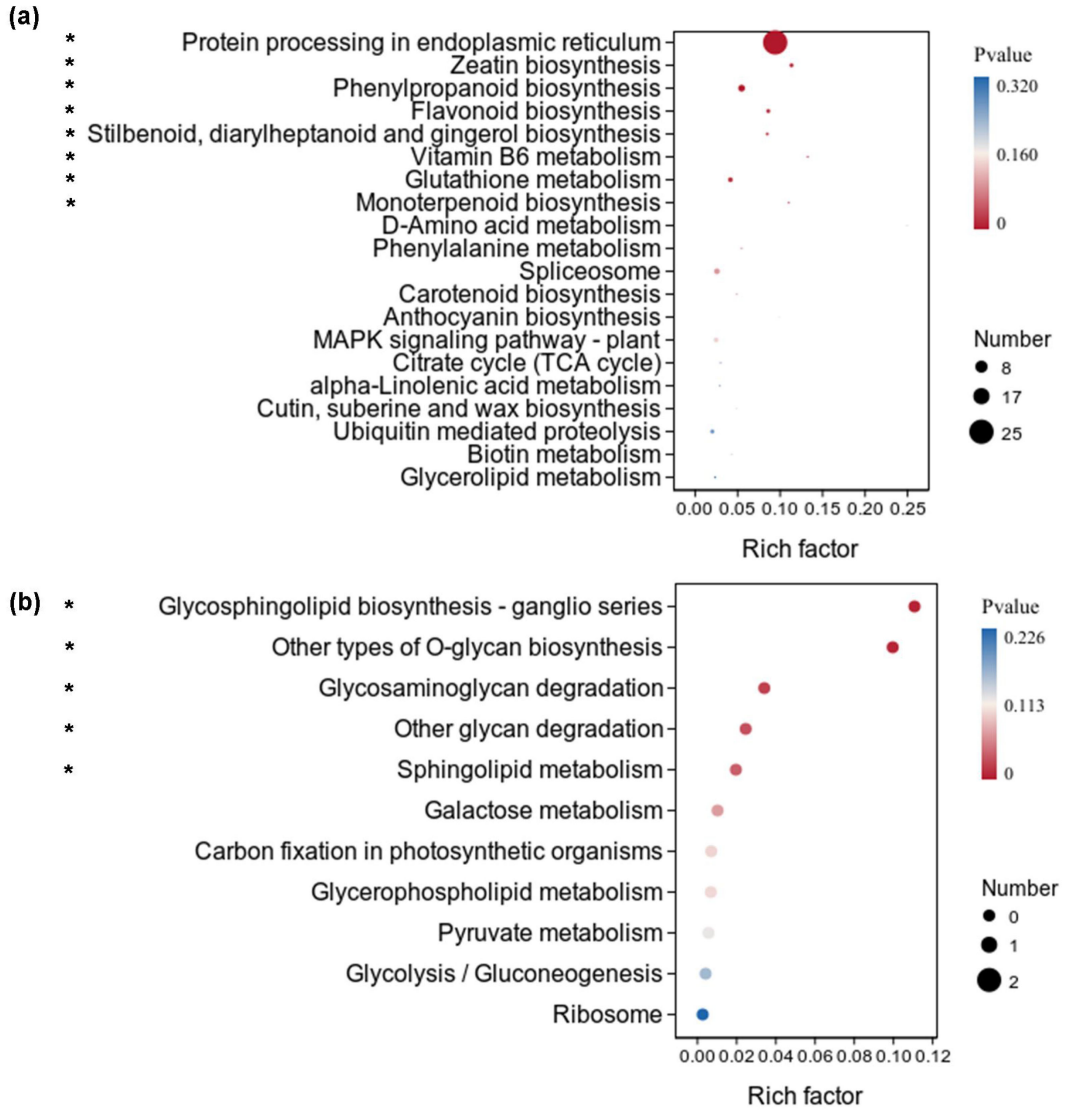
KEGG pathway enrichment analysis of downregulated DEGs under auxin treatment in the aboveground of *X. strumarium***(a)** and *X. sibiricum****(*b*)***. The top 20 KEGG pathways ranked by significance in *X. strumarium* are listed in **Figure 7a**. Downregulated DEGs of *X. sibiricum* were only enriched in 11 KEGG pathways. * indicates that downregulated DEGs were significantly enriched in this KEGG pathway (*P* < 0.05). Different colors represent different *P* values, increasing from red to blue. The sizes of the circle represent the numbers of downregulated DEGs enriched into different KEGG pathways. Rich factors represent the proportions of downregulated DEGs enriched to all genes in this KEGG pathway.

The downregulated DEGs of *X. strumarium* were significantly enriched in eight KEGG pathways, including protein processing in endoplasmic reticulum, zeatin biosynthesis, phenylpropanoid biosynthesis, flavonoid biosynthesis, stilbenoid, diarylheptanoid, and gingerol biosynthesis, vitamin B6 metabolism, glutathione metabolism, and monoterpenoid biosynthesis (**Figure 7a**). The significance and numbers of DEGs enriched in protein processing in endoplasmic reticulum were the highest. Many ubiquitin-dependent protein degradation-related genes are significantly inhibited by auxin (**Supplementary Figure S3**), increasing protein usage time and reducing resource waste. The rich factors of DEGs enriched in vitamin B6 metabolism were the highest (**Figure 7a**). Interestingly, the downregulated DEGs of *X. strumarium* were significantly enriched in some defense and adversity resistance-related KEGG pathways, such as flavonoid biosynthesis and glutathione metabolism. However, the downregulated DEGs of *X. sibiricum* were not significantly enriched in flavonoid biosynthesis (**Figure 7b**). Conversely, the upregulated DEGs of *X. sibiricum* were enriched in flavonoid biosynthesis, although not significant (**Figure 6b**). *X. strumarium* and *X. sibiricum* adopted different growth defense strategies in response to auxin. There were five KEGG pathways which the downregulated DEGs of *X. sibiricum* were significantly enriched in (**Figure 7b**). However, the numbers of DEGs enriched in these KEGG pathways were too small (only one for each KEGG pathways) to reflect the auxin responsive mechanism of *X. sibiricum*.

### The metabolite synthesis responses to auxin in *X. strumarium* and *X. sibiricum*

**Figure 8:**
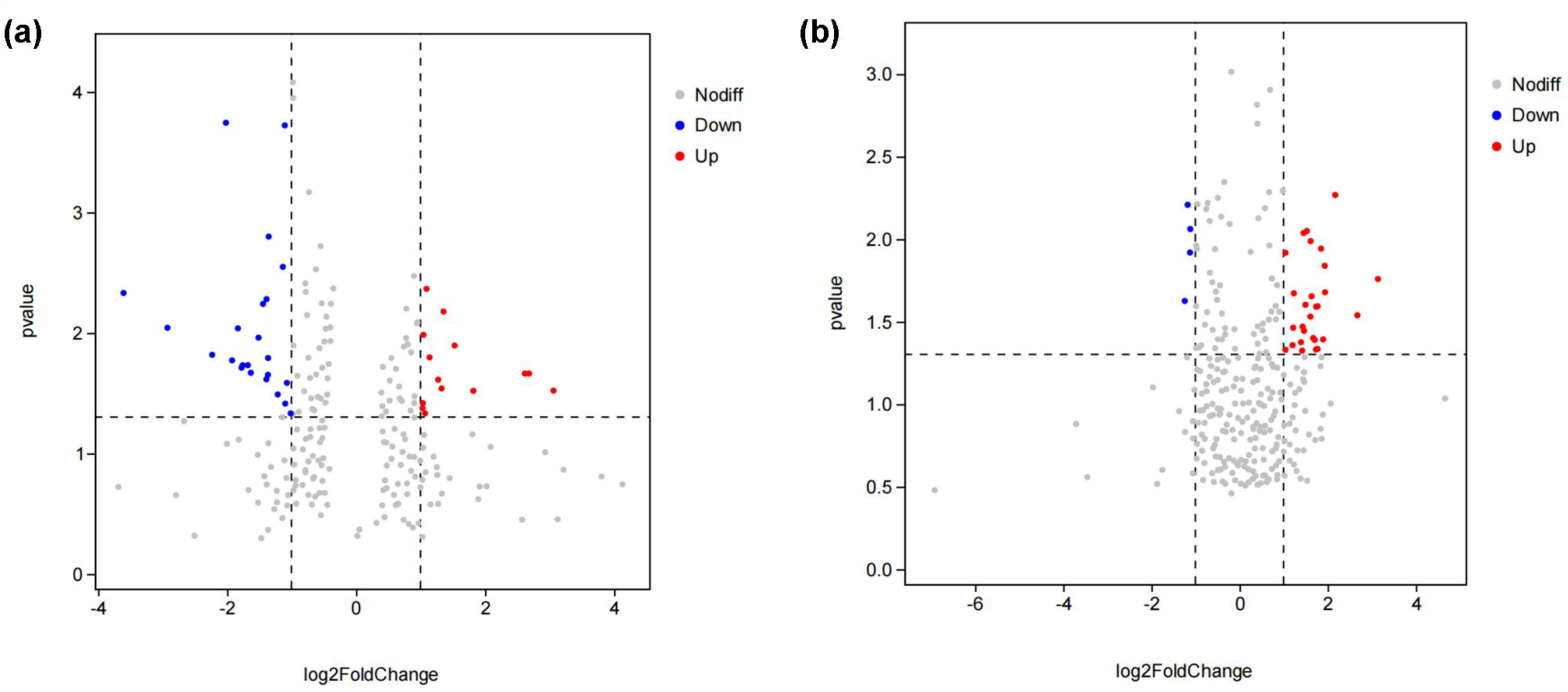
Volcano maps of differential accumulated metabolites (DAMs) between auxin treatment and control group in the aboveground of *X. strumarium***(a)** and *X. sibiricum****(*b*)***. The red, blue, and gray dots represent significant increases, decreases, and no significant changes in metabolite content under auxin treatment, respectively.

To explore the mechanism of auxin promoting the growth of *X. strumarium* at the metabolic level, and compare the differential response to auxin between *X. strumarium* and *X. sibiricum* at the metabolic level, metabolomic analysis was also performed. A total of 1,157 and 1,578 metabolites were detected in *X. strumarium* and *X. sibiricum*, respectively (**Figure 8**). The proportions of lipids and lipid-like molecules were the highest in two kinds of *Xanthium* in the positive detection modes, followed by organoheterocyclic compounds (**Supplementary Figure S4**). The proportions of lipids and lipid-like molecules were also the highest in two kinds of *Xanthium* in the negative detection modes. For *X. strumarium*, the numbers of metabolites belonging to organic oxygen compounds are the second highest in the negative detection modes. Unlike *X. strumarium*, the second most abundant metabolites in *X. sibiricum* were organic acids and derivatives in the negative detection modes.

**Figure 9:**
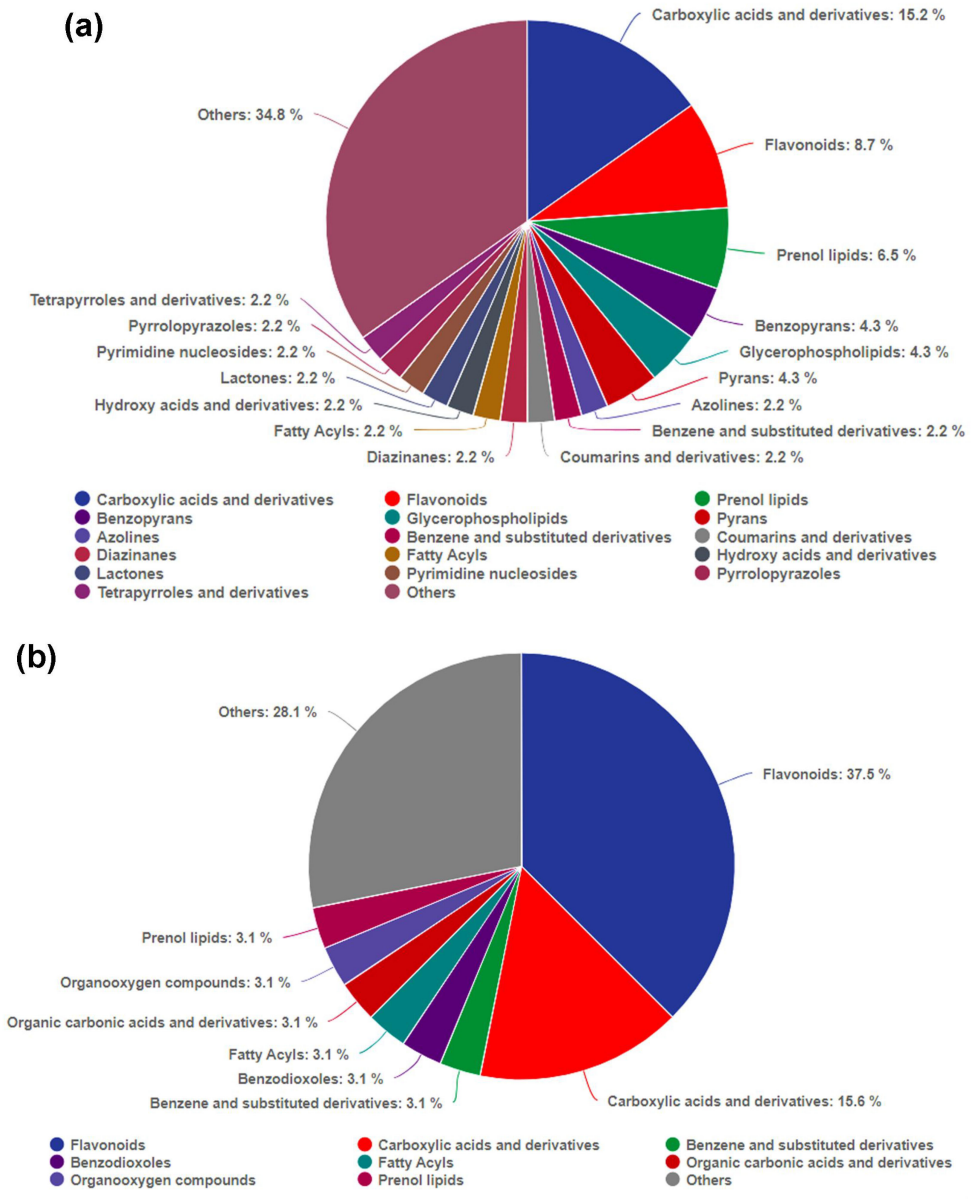
Classes of DAMs between auxin treatment and control groups, and their proportions in all DAMs in the aboveground of *X. strumarium***(a)** and *X. sibiricum****(*b*)***. Different colors represent different classes of DAMs.

**Figure 10:**
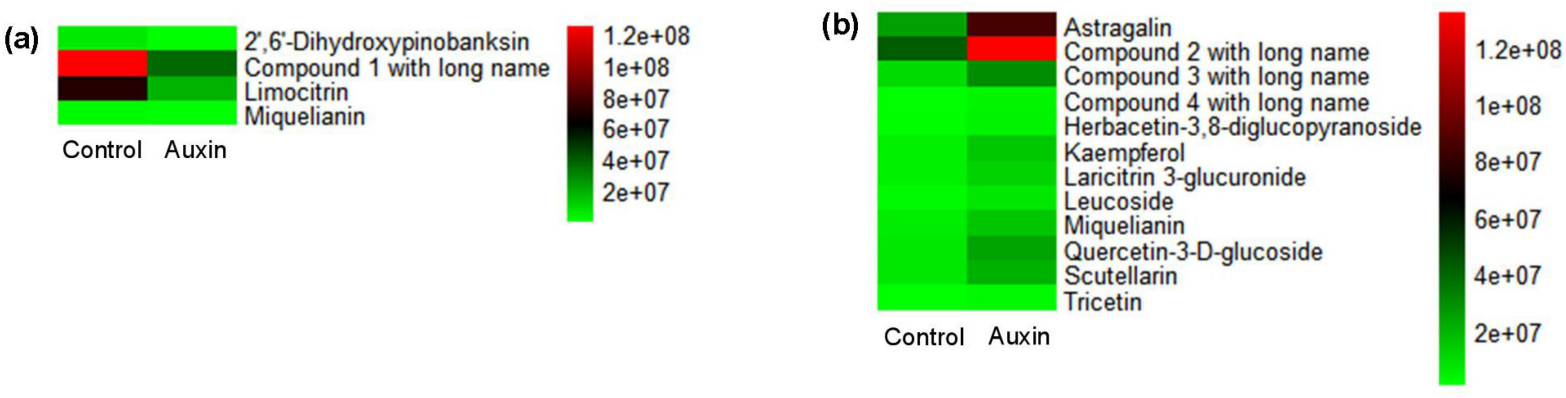
Abundance values of flavonoids with differential accumulation between auxin treatment and control group in the aboveground of *X. strumarium***(a)** and *X. sibiricum****(*b*)***. Different colors represent different abundance values, increasing from green to red. Compounds 1, 2, 3, and 4 with long names refer to 2-(3,4-dihydroxyphenyl)-5,7-dihydroxy-6,8-dimethoxy-4H-chromen-4-one; 5,7-dihydroxy-2-(4-hydroxyphenyl)-3-[(2S,3R,4S,5S,6R)-3,4,5-trihydroxy-6-(hydroxymethyl)oxan-2-yl]oxychromen-4-one; 2-(3,4-dihydroxyphenyl)-5-hydroxy-4-oxo-4H-chromen-7-ylbeta-D-glucopyranosiduronic acid; and 5,7-dihydroxy-2-(4-hydroxyphenyl)-3-[(2S,3R,4S,5S,6R)-3,4,5-trihydroxy-6-[[(2S,3R,4S,5S)-3,4,5-trihydroxyoxan-2-yl]oxymethyl]oxan-2-yl]oxychromen-4-one, respectively.

There were 46 and 32 differential accumulated metabolites (DAMs) in *X. strumarium* and *X. sibiricum* between auxin treatment and control, respectively (**Figure 8**). Most of DAMs in *X. strumarium* showed a significant decrease in content under auxin treatment, whereas most of DAMs in *X. sibiricum* showed a significant increase. The DAMs in *X. strumarium* had the highest proportion in carboxylic acids and derivatives, followed by flavonoids (**Figure 9**). The DAMs in *X. sibiricum* had the highest proportion in flavonoids, followed by carboxylic acids and derivatives. All differential accumulated flavonoids in *X. strumarium* were suppressed by auxin, whereas all differential accumulated flavonoids in *X. sibiricum* were induced by auxin (**Figure 10**), supporting transcriptomic analysis results. *X. strumarium* tended to reduce the accumulation of defensive substances (such as flavonoids) in the response to auxin, whereas *X. sibiricum* took the opposite approach.

## Discussion

Consistent with our hypothesis, auxin promoted the growth of invasive plant *X. strumarium* and the promoting effect of the same level auxin on the growth of *X. strumarium* was stronger than native plant *X. sibiricum* (**Figure 1**). Furthermore, the auxin concentration was higher in *X. strumarium* than that in *X. sibiricum* (**Figure 2**), which contributed to the growth advantage establishment of *X. strumarium* and promotes its invasion. Recently, the roles of hormones in biological invasion has gradually attracted the attention of ecologists (Chen et al., 2024; Dai et al., 2016; Manoharan et al., 2019; Qi et al., 2022; Shan et al., 2024; Zhang et al., 2023, 2024, 2025). However, most studies only compare the differences in hormone levels between native and invasive species, or the changes in hormone levels of invasive species in different invasion habitats and stages. Few studies compare the differences in the effects of hormones on invasive and native species. Its mechanism is still unclear. Our study is the first one to investigate the differential mechanisms of hormone effects on invasive and native species.

The functions of hormones in the growth and adversity adaptation of invasive plants are the basis for driving invasion. However, related research is extremely lacking. Our study confirmed that auxin had a promoting effect on the growth of *X. strumarium* (**Figure 1**). Exogenous application of auxin significantly increased the photosynthetic rate, leaf area, plant height, stem diameter, and aboveground biomass of *X. strumarium*. Dai et al. (2016) found that exogenous application of GA_3_ significantly increased the relative root and shoot length in invasive plant *Wedelia trilobata*. Qi et al. (2022) also verified the growth-promoting effect of GA using another invasive plant *A. philoxeroides*. However, the functions of other hormones in different invasive plants have not been experimentally validated, which needs further study.

Invasive plants benefit more from hormones due to their higher hormone concentrations compared with native plants (**Figure 2**; Chen et al., 2024; Dai et al., 2016; Manoharan et al., 2019; Zhang et al., 2023, 2024, 2025). Our study showed that the concentrations of growth-promoting hormone auxin were significantly higher in invasive plant *X. strumarium* relative to native plant (**Figure 2**), effectively promoting its growth. The concentrations of another growth-promoting hormone GA_1 + 3_ were also significantly higher in invasive plant *W. trilobata* compared with native plant *W. chinensis* (Dai et al., 2016). The concentrations of adversity-resistant hormone ABA were significantly higher in invasive plants *S. trilobata* than its native plant *S. calendulacea* (Zhang et al., 2024). The relative water content, soluble sugar content, and enzyme activity of CAT, SOD, and POD were significantly higher in invader relative to native plant, which promoted its invasion in drought habitats. The concentrations of defensive hormone JA were significantly higher in invasive plant *A. philoxeroides* relative to native plant *A. sessilis* (Manoharan et al., 2019). The cell death area was significantly lower in the invader than native plant, which promoted the invasion of *A. philoxeroides* in habitats with pathogenic bacteria present. Moreover, the concentrations of some hormone in invasive plants were also changed at different parts and scenes (Chen et al., 2024; Zhang et al., 2024). Invasive plant *M. micrantha* invade by climbing and covering trees. The concentrations of auxin, cytokinin, and GA were significantly higher at the turning part of the stem relative to the upper part, which contributed to quickly covering trees and promoting invasion (Chen et al., 2024). The concentrations of IAA and GA_3_ were increased, and the concentrations of JA and SA were decreased in invasive plant *F. bidentis* when it competed with local plants, which contributed to quickly establishing growth advantages over local plants and promoting invasion (Zhang et al., 2024).

In addition to the higher hormone contents, the stronger effect of hormones on invasive plants relative to native plants is also an important reason for hormone-driven plant invasion. However, few studies have focused on it. Our study found that the same level of auxin has a stronger promoting effect on the growth of *X. strumarium* compared with *X. sibiricum* (**Figure 1**). On the one hand, the invader had a stronger activation of auxin signal transduction in response to auxin compared with native congener (**Figures 4**, **6**, **Supplementary Figure S2**), which generated more differential expression genes (**Figure 3**). On the other hand, auxin had different effects on the growth defense strategies of *X. strumarium* and *X. sibiricum* (**Figures 4**–**7**, **10**). *X. strumarium* tended to reduce resource investment in resistance to adversity and defense under the action of auxin, whereas *X. sibiricum* do not, which contributed to the strong growth promoting effects of auxin on *X. strumarium* (**Figure 1**).

The resource allocation between growth and defense plays an important role in the successful invasion of invasive plants (Liu et al., 2022b; Zhang et al., 2024). Our results showed that auxin inhibited many flavonoid—a kind of defensive substance—synthesis-related genes in *X. strumarium* (**Figure 7a**). However, auxin induced many flavonoid synthesis-related genes in *X. sibiricum* (**Figure 6b**). As a result, all differential accumulated flavonoids were suppressed by auxin in *strumarium* (**Figure 10a**), whereas all differential accumulated flavonoids were increased under auxin (**Figure 10b**). In addition, many stress response genes were inhibited by auxin in *X. strumarium* (**Figure 5a**), whereas downregulated DEGs were not significantly enriched in the stress response-related GO terms of *X. sibiricum* (**Figure 5b**). More resources were used for growth in *X. strumarium* under the action of auxin, promoting its successful invasion. It was reported that more resources were also allocated to competitive traits from defensive traits in invasive plant *Spartina alterniflora* when it was introduced into China from the original habitat, which contributed to establishing growth advantages over native plants and thus promoted its invasion (Liu et al., 2022b). Changes in the concentrations of growth-promoting hormones and defensive hormones were involved in the allocation of resources between growth and defense (Zhang et al., 2024). In addition to auxin, GA_3_ has also been reported to have different promoting effects on the growth of invasive and native plants (Dai et al., 2016). However, the mechanism of the differential effects of GA_3_ on the growth of *W. trilobata* and *W. chinensis* was still unclear. More studies about the differences in the effects of hormones on invasive and native plants and their mechanisms are needed.

Photosynthesis plays an important role in the successful invasion of invasive plants (Cai et al., 2023; Feng, 2008; Liu et al., 2022a; van Kleunen et al., 2010). A meta-analysis based on 125 invaders and 196 native plants showed that the invaders generally had higher photosynthetic rates than native plants (van Kleunen et al., 2010). Liu et al. (2022a) obtained the similar results through investigating 97 pairs of invasive and native plants. Compared with associated vines, invasive plant *M. micrantha* had a higher net photosynthetic rate, which promoted its successful invasion (Cai et al., 2023). Our previous study found that *X. strumarium* had a higher photosynthetic rate than *X. sibiricum* (Zhang et al., 2023). However, the reason why invasive plants have strong photosynthetic rate is still unclear. In this study, we found that auxin enhanced the photosynthetic rate and upregulated DEGs under auxin treatment were significantly enriched in many carbohydrate metabolism related pathways in *X. strumarium* (**Figures 1**, **6**). Higher auxin concentrations were found in *X. strumarium* relative to *X. sibiricum* (**Figure 2**), which was one of the reasons for the higher photosynthetic rates in *X. strumarium*.

Auxin also significantly induced the expression of many cell growth- and division-related genes to better utilize resources increased by reduced defense investment and enhanced photosynthesis in *X. strumarium*, whereas upregulated DEGs under auxin treatment were not significantly enriched in the cell growth- and division-related GO terms of *X. sibiricum* (**Figure 4**). As a result, the promoting effect of auxin on plant height, stem diameter, leaf area, and aboveground biomass was significantly higher in *X. strumarium* than those in *X. sibiricum* (**Figure 1**). Moreover, the increased leaf area in *X. strumarium* could further enhance its photosynthesis, thereby expanding its growth advantage over native plants and promoting its successful invasion.

Finally, the hormone-driven hypothesis are proposed based on the results obtained from our study and relevant research on the roles of hormones in invasion. However, more studies are still needed to verify it. The key points are as follows: 1) Plant hormones can promote growth, resist adverse environmental factors, and defend against pests and pathogens in invasive plants. 2) The concentrations of certain functional hormones in invasive plants are higher than those in native plants, including the original concentrations of hormones being higher than those in native plants and the hormone concentrations changing faster than those in native plants in certain special environments and invasion stages. 3) There are differences in the effects of hormones on some physiological processes between invasive and native plants, including differences in strength and opposite effects, leading to stronger promotion of growth, stress resistance, and pathogen and pest defense in invasive plants.

## Conclusion

Auxin promotes the growth of *X. strumarium* by enhancing photosynthesis, reducing resource allocation toward defense and stress resistance, and promoting cell division and growth. Unlike *X. strumarium*, auxin only enhances the photosynthesis of *X. sibiricum*, does not alter the resource allocation toward defense and stress resistance, and does not promote cell division and growth, which results in the stronger growth-promoting effect of auxin on *X. strumarium* than *X. sibiricum* at the same level of auxin. In addition, *X. strumarium* has a higher auxin concentration relative to *X. sibiricum*, further enhancing its growth advantage over *X. sibiricum* and promoting its successful invasion.

## Data Availability

The data presented in this study are publicly available. The data can be found here: https://www.ncbi.nlm.nih.gov, accession PRJNA1303975.
