# Supplementary material for: The mechanism of auxin driving Xanthium strumarium invasion
Source: Front Plant Sci. 2025 Nov 27;16:1705498. doi: 10.3389/fpls.2025.1705498 (PMC12695812; doi:10.3389/fpls.2025.1705498)
Supplement: Supplementary file 1 [file SupplementaryFile1.docx]

**Table S1** Statistics of the read alignments in RNA-Seq study

Sample Reads Bases N (%) Q20 (%) Q30 (%)

Ic-1 4.20 ×10^7^ 6.34 ×10^9^ 0.02 98.93 96.96

Ic-2 7.16 ×10^7^ 1.08 ×10^10^ 0.02 99.01 97.17

Ic-3 4.11 ×10^7^ 6.21 ×10^9^ 0.02 99.09 97.37

Ia-1 4.25 ×10^7^ 6.42 ×10^9^ 0.02 98.97 97.07

Ia-2 4.88 ×10^7^ 7.37 ×10^9^ 0.02 98.90 96.91

Ia-3 4.43 ×10^7^ 6.68 ×10^9^ 0.02 99.02 97.20

Nc-1 4.31 ×10^7^ 6.51 ×10^9^ 0.10 98.79 96.71

Nc-2 4.62 ×10^7^ 6.76 ×10^9^ 0.10 98.85 96.90

Nc-3 4.58 ×10^7^ 6.92 ×10^9^ 0.10 98.83 96.90

Na-1 4.20 ×10^7^ 6.35 ×10^9^ 0.10 98.80 96.77

Na-2 4.81 ×10^7^ 7.27 ×10^9^ 0.10 98.90 97.01

Na-3 4.07 ×10^7^ 6.14 ×10^9^ 0.10 98.88 96.89

I and N represent invasive plant *X. strumarium* and native plant *X. sibiricum*, respectively. c and a represent control and auxin treatment, respectively. The numbers (1, 2 and 3) represent three independent biological replicates for each treatment.


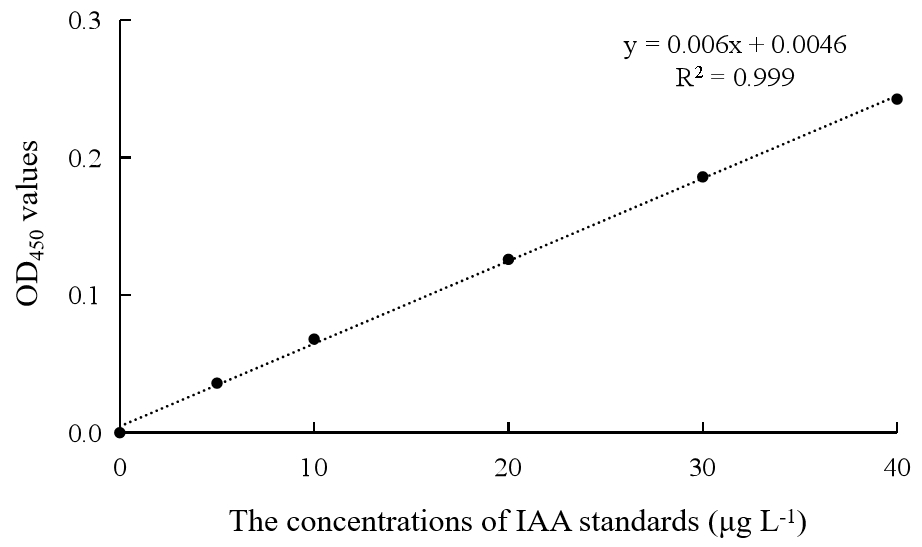


**Fig. S1**

Standard curve for IAA content determination using ELISA kit. 80 μg L^-1^ IAA standard was provided in plant IAA ELISA kit and was gradient diluted to 40, 30, 20, 10 and 5 μg L^-1^, respectively. The concentrations of these IAA standards were measured under 450 nm.


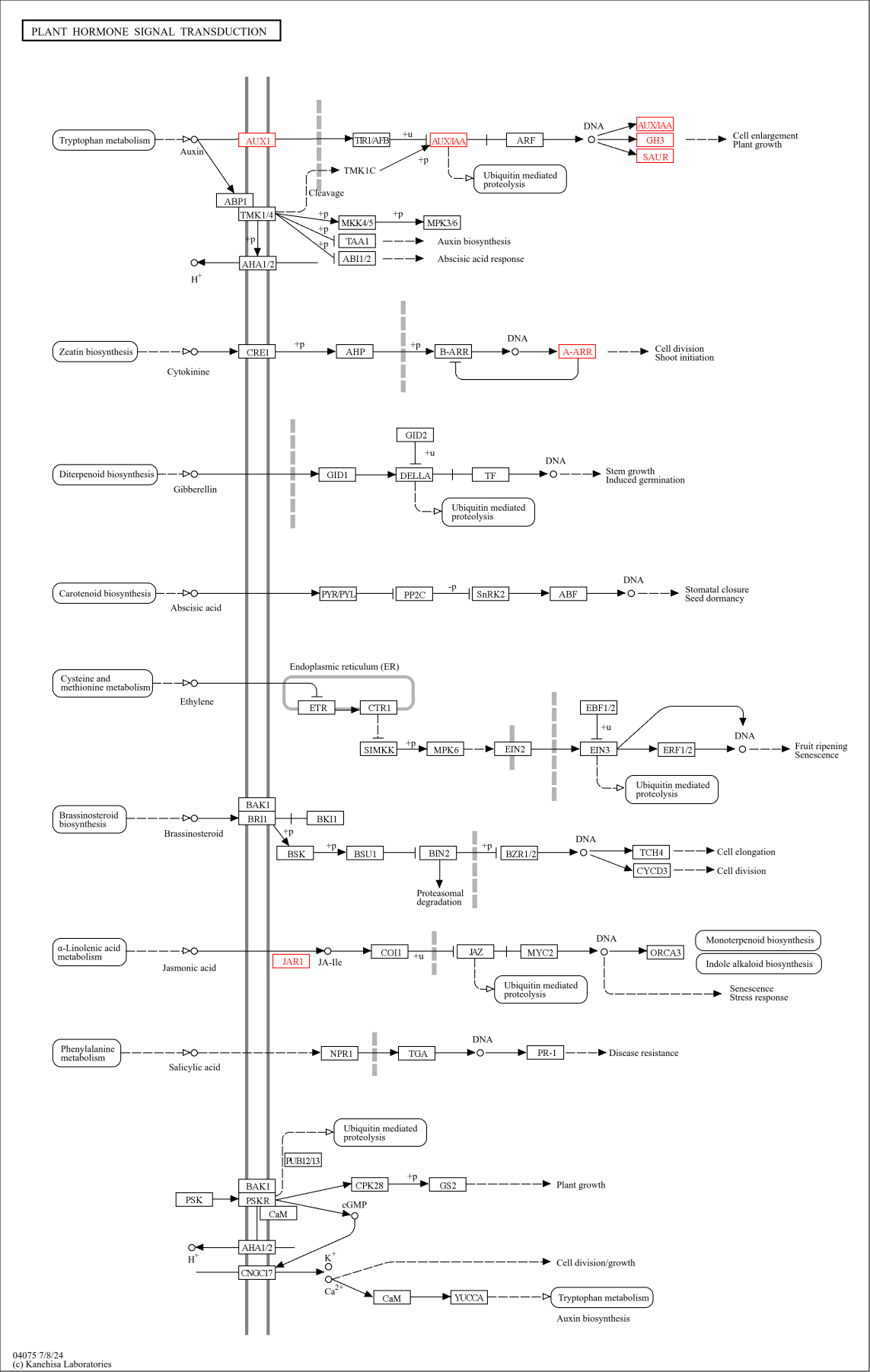


**Fig. S2**

Enrichment of up-regulated DEGs under auxin in plant hormone signaling pathways in the above ground part of *X. strumarium*. Red labels indicate that these DEGs enriched in plant hormone signaling pathways are induced by auxin.

**
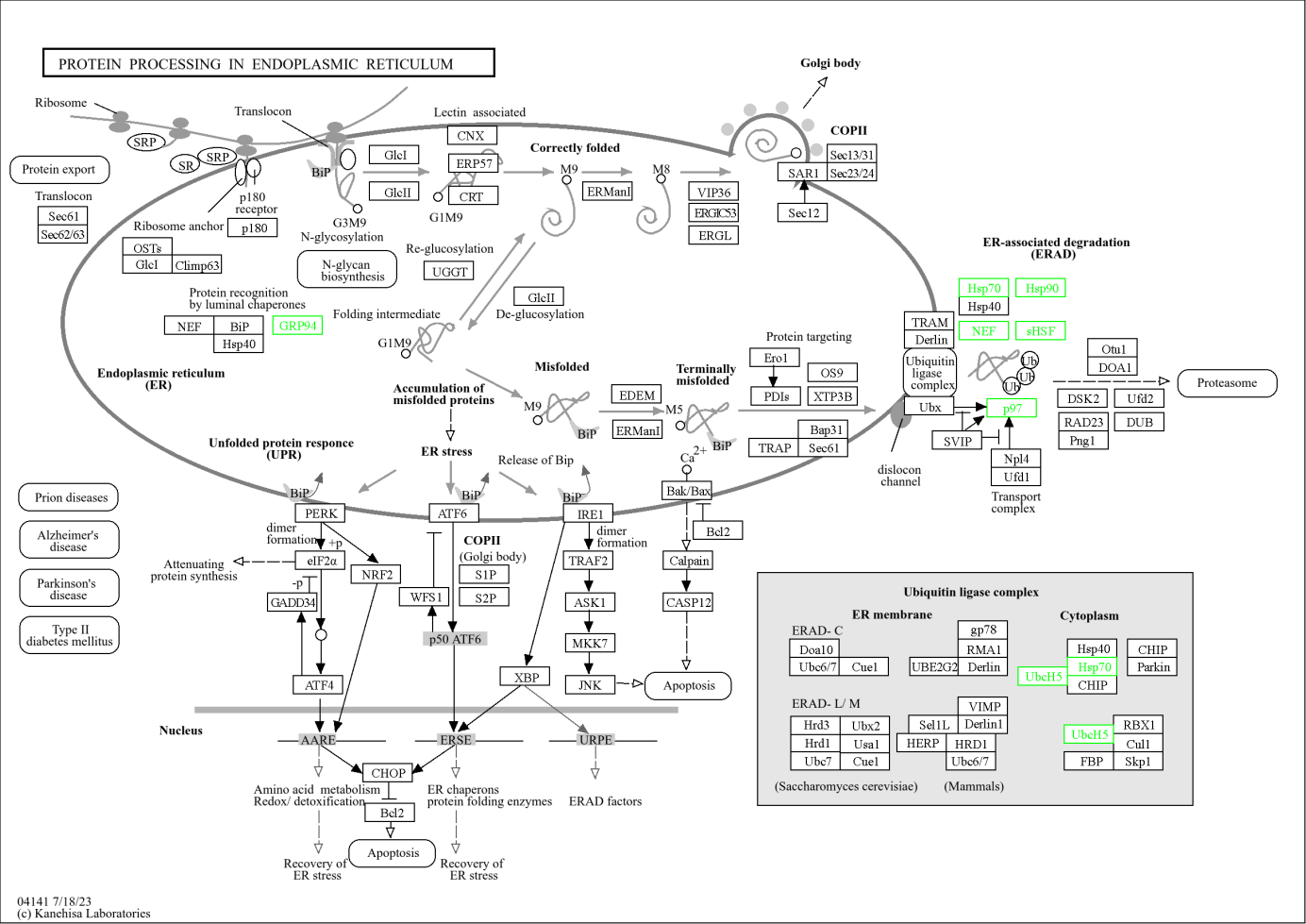
**

**Fig. S3**

Enrichment of down-regulated DEGs under auxin in protein processing in endoplasmic reticulum in the above ground part of *X. strumarium*. Green labels indicate that these DEGs enriched in protein processing in endoplasmic reticulum are inhibited by auxin.

**Fig. S4**

Metabolite identification in the aboveground of *X. strumarium* (a, b) and *X. sibiricum* (c, d) in positive (a, c) and negative (b, d) ion mode. The proportions of different classes of DAMs were displayed as pie charts.
